# A ten-year historic study of paranasal cavity endoscopy in patients with leprosy

**DOI:** 10.1016/S1808-8694(15)31265-9

**Published:** 2015-10-20

**Authors:** Ana C.C. Martins, Jair de Carvalho e Castro, João Soares Moreira

**Affiliations:** 1 Ph.D. studies in Dermatology under course, Federal University of Rio de Janeiro – UFRJ. Otorhinolaryngologist, Instituto de Pesquisa Clínica Evandro Chagas – Fiocruz.; 2 Ph.D., Professor in Otorhinolaryngology, Federal University of Rio de Janeiro- UFRJ.; 3 Physician and Full Researcher in Otorhinolaryngology, Instituto de Pesquisa Clínica Evandro Chagas – Fiocruz.

**Keywords:** leprosy, nose leprosy, nasal endoscopy

## Abstract

Leprosy is an infectious condition that has a chronic evolution caused by the *Mycobacterium leprae*. It very often attacks the nasal cavities mucosa independent of its clinical form, even before skin lesions or lesions to other parts of the body arise, in the presence or not of clinical complaints. **Aim:** To show the efficiency of nasal endoscopy to identify endonasal mucosa lesions and the importance of the Otorhinolaryngologist in the diagnosis and follow-up of Leprosy patients. **Study Design:** Clinical history study. **Material and Method:** a historic study was performed with 173 patient’s records without previous treatment from 1990 to 2000 at the Otorhinolaryngology Services, Instituto de Pesquisas Clinicas Hospital Evandro Chagas, Fiocruz. **Results:** All of the patients showed nasal lesions, 121 with and 52 without clinical complaints. **Discussion:** Nasal cavities endoscopy exam enabled early identification of the mucosa alteration in Leprosy patients as well as how to identify the evolution of the lesions. This type of exam also helps to establish local treatment. **Conclusion:** The evaluation and follow-up of Leprosy patients by the Otorhinolaryngologist in a multidisciplinary team are justified and offer the patient early diagnosis and specific treatment.

## INTRODUCTION

The agent that causes Leprosy is a bacillus named *Mycobacterium leprae* or Hansen bacillus, which belongs to the family of Mycobacterium, which include many different microorganisms that cause or not disease in humans. It is alcohol acid-resistant, does not get discolored with alcohol and acids and is stained by acid fucsin in red ^1^. It is an obligatory intracellular parasite, presenting affinity with skin cells and peripheral nerve cells. Hansen bacillus reproduces slowly. Its average time of multiplication is 12 to 14 days, very long if compared to tuberculosis bacillus, which takes 20 hours. This biological slowness of the bacillus explains its chronic progression and the long period of incubation, which occurs on average from 2 to 5 years. Humans are considered the only natural reservoir for disease transmission; however, some animals such as chimpanzees (*Pan troglodytes*), mangabei monkey (*Dercocebus torquatus atys*) and armadillo (*Dasypus novencintus*) could be probable reservoirs of the infection, given that in these three species leprosy is naturally acquired ^2^. According to Lombardi et al. (1990) ^3^, the social environment seems to be important in the transmission of the disease, and there is no question about the correlation between poverty and risk of leprosy, with clear delimitation of the more susceptible group among low-income populations, but not knowing whether this factor is related with nutritional status, home agglomeration or presence of other concomitant diseases, explaining the endemics of the disease. Leprosy may affect both genders, and it finds more cases in men, but there is no explanation to this fact. The initial reason for the present paper was the fact that there are few studies published in the literature about the presence of lesions in paranasal sinuses mucosa, observed through endoscopic study performed by Otorhinolaryngologists. The use of optical fiber as a routine may increase the level of success in locating and identifying mucosa affections that occur in leprosy patients.

## LITERATURE REVIEW

Chacko et al. (1979) ^4^ described that *Mycobacterium leprae* may be found in many different parts of the body surface, such as skin of the extremities, earlobes, helix, antihelix, tragus, alar cartilage, septum and testicles. The temperature in these areas is lower than the normal body temperature, providing a favorable environment for the bacillus. Multibacilli untreated patients are a source of infection, whereas patients with paucibacillary and undetermined forms, with negative bacilloscopy, in addition to infected and asymptomatic people, may have an important role in the transmission of the disease.

The transmission of the disease occurs by direct contagious through prolonged contact between untreated multibacilli patients and non-infected subjects, even though there is the unconfirmed possibility that it is processed through indirect methods, with contaminated objects and/or vectors. 3, 5, 6, 7

We can also note the importance of nasal mucous in the transmission of the infection, which may be a primordial factor for the dissemination of the disease, but other paths may be involved. The nasal mucosa is considered as entry and exit doors for *Mycobacterium leprae* in many cases considering how vulnerable the openings are and the free access to the bacilli. 3, 7, 8, 9

It is admitted that inoculation can happen through the nasal mucosa and by means of continuity solutions, such as damaged nodules on the skin, breast milk, as well as cuts and scratches on the skin of patients that may be transmissions routes, with incubation period that varies from 2 to 5 years, on average. Predisposed patients may have family contacts with patients that have multibacillary forms, extra-home contacts with multibacilli patients and contact with patients that have the paucibacillary form.

There is no uniformity in relation to disease distribution, but the areas of higher prevalence are in less developed regions. An important fact concerning Leprosy is that most endemic countries are located in tropical and subtropical weather, where there are low social-economic levels, people living in poor housing, nutritional and hygiene conditions, with poor medical support and ineffective preventive control of the disease; Brazil ranks second in leprosy, behind India ^10^.

Leprosy is a public health problem in developing countries and the magnitude of Hansen’s endemics is the number of existing cases in a specific area and those that are recorded annually, either the absolute number of cases in a given moment or diagnosed in the year, or the relative number, which is the coefficient of prevalence or records. Brazil has an agreed goal with WHO to reduce the prevalence rate to less than one case of Leprosy per 10,000 inhabitants by 2005.^3^, ^6^, ^7^, ^8^, ^9^, ^10^, ^11^

The diagnosis is made by clinical history and dermatological-neurological exam, complemented by tests and laboratory exams, such as bacilloscopic examination, performed in all patients with clinical suspicion of the condition. The sites of material collection are areas that have sensitivity affection or active lesions, such as smear of active lesion or area with sensitivity abnormalities. In the absence of lesions or numb areas, the collection is made through the smear of four sites: two auricular lobes and two elbows, to be stained with Ziehl-Neelsen solution. 1, 2, 10

Many authors are unanimous about the presence of bacillus in the nasal mucus, referring to the diagnostic value of the exam, as well as to the prophylactic profile, associated with clinical examination and investigation of bacilli in the skin lesions. The mucus is positive in a high percentage of patients with Lepromatous and Borderline leprosy, provided that repeated exams are performed, given that they may sometimes be negative.

According to Cerruti et al. (1945) ^12^, in the Neural form, the mucus exam does not allow early diagnosis, given that the positivity occurs after the evidence of other symptoms, either dermatological or neurological, sufficient to lead to the diagnosis.

The clinical presentations of leprosy comply with the spectral classification by Ridley and Jopling, from the 60’s, in which we can find Tuberculoid form, polar stable (TT), with negative bacilloscopy, major cell response, limited lesions and few bacilli; LL form (polar Lepromatous or Virchowian form), pole of high sensitivity to *M. leprae,* with skin lesions that are diffusely distributed on the skin and exacerbated humoral response, and the forms from the Borderline group (unstable): BT (paucibacillary), BB and BL (multibacillary). It is important to mention the pure Neural form (few bacilli) and the Undetermined form (I), responsible for the initial manifestation of the disease, in which the host response is insufficiently differentiated to enable classification, which may evolve to spontaneous cure or polarization, depending on the capacity to form cell immune response against *M. leprae.*

The chronic progression of Leprosy may be manifested sometimes by acute phenomena, which are named reaction episodes, related with the immune status of the subject; they may be present both in the treatment or after discharge and they do not require suspension or restart of polychemotherapy. Reaction episodes are types I, II and Neural. Type I reactions are mediated by cells (cell immunity) and occur in paucibacillary patients. Type II reactions are mediated by antibodies (humoral immunity) 2, 3, 4, 5, 6, 7, 8, 9, 10 and occur in forms LL and BL.

Treatment is mainly in outpatient settings and primary health care centers use an association of drugs, standard polychemotherapy OMS (PQT/OMS), using Rifampicin, Dapsone and Clofazimine. Treatment course varies from six months to one year. After two weeks from onset of treatment, the Hansen patient is not longer infecting. 2, 3, 4, 5, 6, 7, 8, 9, 10

As to nasal manifestations of the disease, Leloir (1886) ^13^ referred to the affection of nasal, mouth, throat, larynx and eye mucosa, which could be invaded since the beginning of skin tuberous eruption, especially of the nose.

As years went by, many publications unanimously confirmed the nose as the initial site of Hansen’s lesions. 4, 5, 11, 12, 13, 14, 15, 16, 17, 18, 19

The nasal mucosa will be considered affected when we evidence, by anterior and posterior rhinoscopy, typical and individualized lesions of leprous rhinitis, such as infiltration, lepromas, ulceration and perforation, as well as other observed aspects that are not part of leprous rhinitis, such as discoloration or paleness of the mucosa, congestion, ectasias, vasculitis, crusts, atrophy, dryness and presence of blood, which may be found in other nasal affections. In many patients, however, these aspects were determined by the leprous infections, as demonstrated by the bacterioscopy, and especially at the histopathology, evidencing the perivascular and neural infiltration specific to Leprosy. 5, 12, 20

The performance of ENT examination as a routine in Hansen’s patients, using nasal speculum or rigid and flexible endoscope, may provide further accuracy for the identification of lesions that had not been visualized before, enabling the diagnosis, in association with clinical history, plus its use to follow up patients so as to prevent sequelae.

## OBJECTIVES

### Specific Objective

To confirm the efficacy of endoscopic examination in the identification of mucosa lesions of paranasal sinuses in patients with Leprosy, regardless of the clinical presentation.

## MATERIAL AND METHODS

After the approval of the project by the Technical and Scientific Medical Ethical Committee (FIOCRUZ), we performed a retrospective study in which we assessed the medical charts of patients with Leprosy of the Service of Otorhinolaryngology, FIOCRUZ, from 1990 to 2000, without previous treatment and regardless of clinical presentation or ENT complaints, and no differentiation between gender, race, age or social class. The patients were seen in the Ambulatory Souza Araújo, FIOCRUZ, responsible for the diagnostic investigation, clinical progression and treatment. They were classified according to number of bacilli (multibacillary or paucibacillary) and clinical presentation by clinical history, sensitivity tests, bacilloscopy and later referred to the Service of Otorhinolaryngology and Endoscopy, Instituto de Pesquisa Clínica Evandro Chagas (IPEC), FIOCRUZ. In the service, the patients were submitted to conventional ENT examination using halogen light photophore, sterile nasal speculum for anterior rhinoscopy, disposable wood spatula for oropharyngoscopy and laryngeal mirrors number six for indirect laryngoscopy, completing the endoscopic study of paranasal sinuses using rigid 30° endoscope. There was no need to use topical anesthesia but some patients required cleaning of nasal cavities with sterile solution at 0.9% to remove crusts, so as to facilitate the identification of lesions during the endoscopic examination.

After the ENT examination (conventional and endoscopic), the collected data were stored according to a protocol that included:

1. Identification (name, age, gender, race);
2. Date of diagnosis;
3. Patient’s complaints;
4. Clinical presentation of the disease: Undetermined (I), Tuberculoid (TT), Borderline tuberculoid (BT), Borderline-borderline (BB), Borderline-lepromatous (BL), Lepromatous lepromatous (LL) and pure Neural (NP);
5. Presence of reaction types I, II or Neural;
6. Endoscopic ENT examination.

## RESULTS

Among the 173 patients we found 53 with LL presentation (30.6%), 39 with BL presentation (22.5%), 39 with BT presentation (22.5%), 29 with BB presentation (16.8%), 7 with I presentation (4.1%), 5 with NP (2.9%) and one with TT (0.6%) (Table 1), showing agreement with many different authors in terms of higher frequency of disease in lepromatous forms. We observed in our study that 70% of the patients presented ENT complaints related to the nose and many patients did not correlate nasal symptoms and Leprosy (Table 1). The most frequent complaints were nasal obstruction found in 94 patients, representing 54.3%, followed by epistaxis in 70 patients, amounting to 40.5%, and elimination of crusts, referred by 50 patients, amounting to 28.9% (Table 2); the same complaints had been listed by many studies. 4, 11, 12, 15, 16, 17, 18, 19, 20, 21

**Table 1:** Relation between clinical presentation and presence or not of nose related complaints.

	Patients with complaint	Patients w/out complaint	Clinical presentation
	48	5	LL
	26	13	BL
	22	17	BT
	**17**	**12**	**BB**
	**5**	**0**	NP
	3	4	I
	0	1	TT

Total	121	52

**Table 2:** Relation between nasal complaints and clinical presentations.

Complaints	NP	LL	BL	BB	BT	TT	I	Total	%
Nasal obstruction	2	40	21	13	14	1	3	94	54.3
Epistaxis	2	38	15	4	10	0	1	70	40.5
Crusts	1	26	12	2	5	1	3	50	28.9
Rhinorrhea	1	21	8	5	6	0	2	43	23.7
Hyposmia	0	7	2	3	1	0	0	13	7.5
Nasal pain	0	6	2	3	2	0	0	13	7.5
Pruritus	0	2	1	2	6	0	0	11	6.4
Headache	0	6	2	1	1	0	0	10	5.7
Sneezing	0	1	1	1	1	0	1	5	2.8
Cacosmia	0	3	0	1	0	0	0	4	2.3
Dryness	0	1	1	0	1	0	0	3	1.7
Use of vasoconstrictor	0	1	0	0	1	0	0	2	1.2
Anosmia	0	1	0	0	0	0	0	1	0.6

Key: Undetermined (I), Tuberculoid (TT), Borderline tuberculoid (BT), Borderline-borderline (BB), Borderline-lepromatous (BL), Lepromatous lepromatous (LL) and pure Neural (NP)

In addition to the complaints previously reported as the most frequent, the patients also reported, in decreasing order: rhinorrhea in 43 patients (23.7%), hyposmia in 13 patients (7.5%), 13 patients reported nasal pain (7.5%), 11 had nasal pruritus (6.4%), 10 had headache (5.7%), five had sneezing (2.8%), three patients reported nasal dryness (1.7%), four had cacosmia (2.3%) and one presented anosmia (0.6%) (Table 2).

## DISCUSSION

We agree with Davey (1974) ^8^ concerning the negligence of the nasal mucosa examination using anterior rhinoscopy, which is not performed as a routine in many treatment centers, despite the knowledge that specialists have that there may be nasal mucosa affections, even in the absence of visible clinical signals or symptoms, as reported by Leloir (1886) ^13^. We also considered that the nose is an important site of entry and elimination of bacilli (*Mycobacterium leprae*), a frequent statement in many studies 1, 5, 8, 9, 12, 16, 17. In addition to mucosa lesions (Table 3) found in untreated patients, we detected persistence of these lesions and even evolution with progressive worsening in patients in treatment and after treatment with polychemotherapy, contradicting some authors as Chacko et al. (1979) ^11^ and Davey (1974) ^8^, which confirmed that bacillary elimination of nasal mucosa occurs faster when compared to the skin owing to the fact that many specialists neglected the nasal mucosa examination. We believe that there are indeed bacilli in the nasal mucosa after treatment, but we question the viability of these bacilli, justifying the fact that we had not performed routine bacilloscopy of nasal swab, because we agree with Cerruti et al. (1945) ^12^ that stated that in contacts and in patients with neural presentation, the result of bacilli in nasal mucus is negative in most cases, requiring repetition of the exam.

**Table 3:** Relation of endoscopic findings and clinical presentations of the disease:

Endoscopic findings	NP	LL	BL	BB	BT	TT	I	Total	%
Infiltration	4	38	31	26	36	1	4	140	80.9
Rugosity	5	16	20	19	18	1	2	81	46.8
Dryness	2	24	11	16	13	1	0	67	38.7
Vasculitis	5	22	10	11	17	1	1	67	38.7
Crusts	1	32	10	1	8	1	1	54	31.2
Paleness	1	12	9	6	17	1	4	50	28.9
Ectasias	4	19	5	8	9	1	1	47	27.2
Hyperemia	2	14	12	1	10	1	1	41	23.7
Ulceration	0	23	6	1	4	0	0	34	19.7
Blood	3	5	5	10	3	0	0	26	15
Atrophy	3	9	4	3	4	0	0	23	13.3
Moisture	0	1	4	3	6	0	1	15	8.7
Secretion	0	4	4	2	3	0	0	13	7.5
Perforation	1	6	1	0	1	0	0	9	5.2
Hansenomas	0	7	1	1	0	0	0	9	5.2
Synechia	0	1	0	0	0	0	0	1	0.6

Legendas: Undetermined (I), Tuberculoid (TT), Borderline tuberculoid (BT), Borderline-borderline (BB), Borderline-lepromatous (BL), Lepromatous lepromatous (LL) and pure Neural (NP)

**Photo 3:**
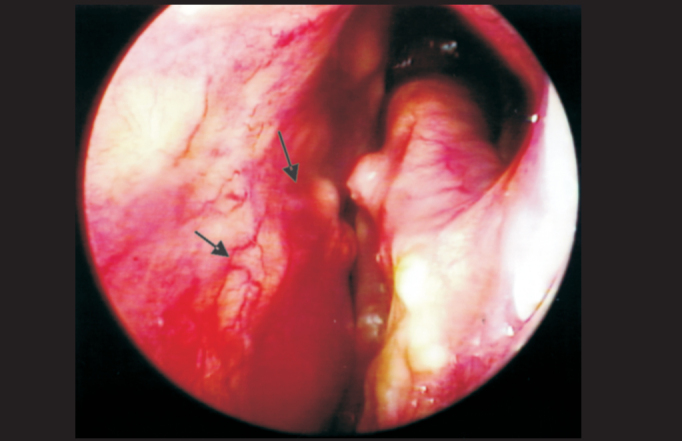
LL presentation with dryness, vasculitis and hansenomas.

We advocate the process to rule out other diseases with Hansen’s signs and symptoms, the performance of nasal biopsy for histopathological exam, even when the dermatological and neurological investigation is negative. We would like to point out that it is possible to suspect of a case of Leprosy only through the clinical history associated with the ENT examination, especially when we perform it with rigid or flexible endoscope, a fact also observed by the authors Soni (1997) ^21^ and Fokkens et al. (1998) ^22^. The exam enables the identification of mucosa lesions that are not observed when we use only nasal speculum in the anterior rhinoscopy, such as hansenomas and vasculitis (Photo 3).

We also observed that 52 patients did not report ENT complaints, a fact observed also by Cerruti et al. (1945)^12^ ; despite the absence of symptoms, these patients presented nasal lesions, justifying the performance of the ENT exam and mainly the endoscopic examination of nasal cavity mucosa in all patients with Leprosy, regardless of the complaints or clinical presentation of the disease.

As to olfaction affections, Barton et al. (1976) ^16^ showed that it is a common complaint, found in 44% of the 150 patients seen with the lepromatous presentations, referring that the affection is related with severity of clinical presentations in the nasal mucosa. Despite the fact that we found a smaller number of patients with olfaction affections relative to previous references, amounting to 7.5% of total hyposmia in assessed patients (Table 2), this complaint was seen not only in patients with lepromatous presentation (LL) - seven cases, but also in patients with BL (two), BB (three) and BT (one) presentations, and these patients were in a more advanced stages of the disease or in a reaction episode, which made us agree with the authors. We would like to point out the fact that this complaint may be found in any clinical presentation of the disease.

Attempts to magnify the mucosa examination were tried with the use of surgical microscope 23, 24, later replaced by telescope for nasal examination. We can observe the identification of lesions using rigid or flexible telescope, which provides characterization of mucosa lesions as suspected of Leprosy, which made us exclude other diseases that were also frequent in our service, such as tuberculosis, leishmaniasis, paracoccidioidomycosis, syphilis, sarcoidosis, rhinoscleroma, ozena and even allergic rhinitis or acute viral rhinitis. We used as a routine the clinical and laboratory investigation of all suspected patients, in addition to not performing exams in the presence of colds or allergic crisis to prevent influence of the results.

Before the introduction of the telescope, some authors^5^, ^16^, ^17^ stated that there were no nasal mucosa lesions in patients with Tuberculoid (TT) or Borderline (BT and BB) presentations, but we found 40 patients with lesions and TT and BT presentations and we noticed that regardless of the clinical presentation, we could found mucosa lesions even in the absence of complaints and at any disease stage.

**Photo 1:**
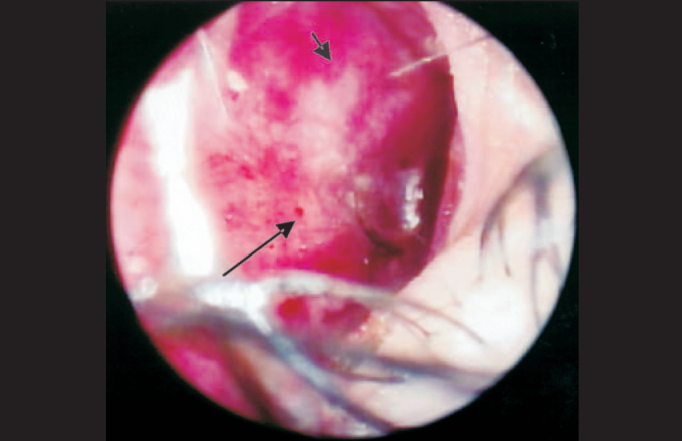
LL presentation with septal anterior-inferior damage, presence of vasculitis, dryness, infiltration, mucosa hyperemia, crusts and blood spots.

We identified nasal mucosa lesions in all assessed patients (100%) (Table 3), a percentage close to that reported in the study by Barton et al. (1976) ^16^ which found early nasal cavity lesions in 95% of the patients with LL and BL presentations. Among the most frequent lesions, we identified nasal mucosa infiltration (Photo 1), defined in the literature 5, 16 as granulomatous infiltration of the mucosa and referred by the patients as nasal obstruction. Even considering the apparent permeability of the nasal cavity, patients may refer nasal obstruction, a common complaint of atrophic leprous rhinitis, associated with reduction of sensitivity, inspiration and expiration perception, initially unilateral and later bilateral, without response to use of topical vasoconstrictor in view of major mucosa infiltration. 5, 8, 16, 22

Fokkens et al. (1998) ^22^ found in 18 out of 40 patients the lepromatous form of infiltration and nasal obstructive complaint. We observed infiltration in 140 patients, representing 80.9% of the total, more frequent in the lepromatous forms (38 patients), as also referred by many other authors. However, our sample is greater, owing to the fact that we could observe mucosa lesions in all clinical presentations of the disease. We found only three patients that used topical vasoconstrictors because of respiratory difficulties. The limited use is probably related with early diagnosis and treatment of these patients by the Service of Otorhinolaryngology, IPEC/ Fiocruz, or the low purchasing power of the population and medication high prices. We think that in patients with damaged mucosa, the use of topical vasoconstrictor could lead to marked worsening of nasal mucosa, meaning that its use is contraindicated as treatment, which could cause superficial ulceration, epistaxis and eventually septal perforation.

In general, lesions are located on the anterior-inferior portions of the nasal cavities, regions appointed by all authors because it provides predisposing factors such as easy access of bacilli, mechanical trauma, low temperature, and others.

**Photo 2:**
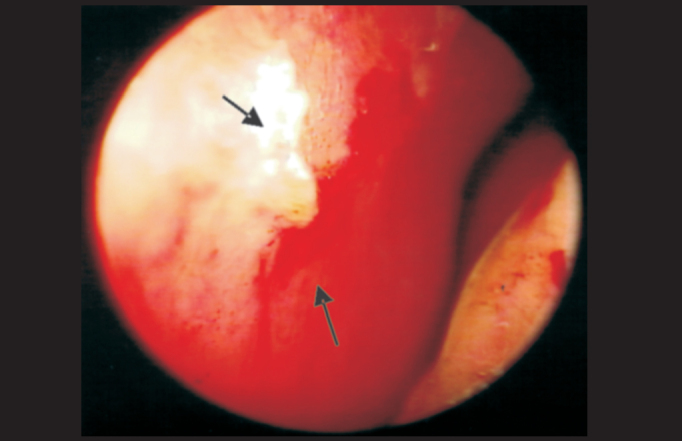
BT presentation with bleeding areas on the septal anterior-inferior region, crusts, and infiltration.

As to epistaxis, we could notice that most cases were related to mechanical trauma and the act of blowing the nose intensively, such as the attempt to remove the crusts 4, 12, 15, 16 or influenced by external factors, such as the topical drugs reported before, inhalation drugs, arterial blood hypertension and hormonal abnormalities, which provide greater capillary fragility, in addition to marked vasculitis, observed especially in the anterior septal region (Kiesselbach area), predisposing to nasal bleeding ^21^ (Photo 2).

**Photo 4:**
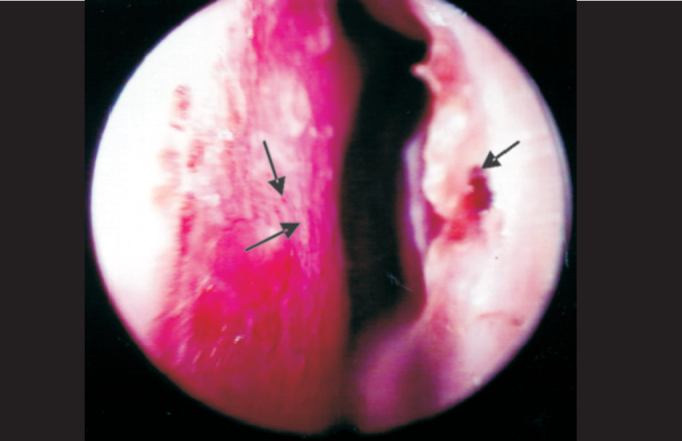
Pure Neural presentation with dryness, hyperemia, vasculitis, crusts and blood.

In addition to infiltration and presence of blood on the mucosa of nasal cavities, we could identify the presence of crusts (Photos 1, 2 and 4) at the nasal endoscopy of 54 patients, representing 31.2% of the 173; it was also a frequent finding in the study by Barton (1974) ^5^, who found it in 74% of the 77 studied patients, by Fokkens et al. (1998) ^22^, who found 62% among the 40 studied patients, and by Srinivasan et al. (1998) ^25^ in 92% of the 25 patients with Lepromatous presentation. Our percentage was smaller when compared to the other studies, but we emphasize that this figure is higher when we analyze only BL and LL presentation, amounting to 24.3% of the 42 studied patients that had it.

Crusts may be found in any clinical presentation, ranging in amount, size, shape, consistency and color and they are comprised by more or fewer leukocytes (purulent crusts), more or less number of red cells (hematic crusts), fibrin, saprophyte germs and epithelial cells at different stages of degeneration. In crusts that cover the ulcerations, in addition to the above-mentioned elements, we can evidence other cell elements, common to infiltrations, followed by necrobiosis of underlying tissues, that may be strongly adhered to septal mucosa and other areas. The presence of crusts is justified by marked dryness of nasal secretion, which takes place during the infectious process by damage to the trigeminal nerve (5th nerve) responsible for parasympathetic innervation, which enables the secretory action of mucosa glands, given that *Mycobacterium leprae* has neural tropism. ^4^

Barton (1974) ^5^ had not observed lesions in patients with Tuberculoid and Neural presentations, stating that the affection of the latter is restricted only to the peripheral nerve system, generating neural thickness and atrophies and sequels. We attracted the attention of specialists because we found considerable frequency of mucosa lesions in these two clinical presentations of the disease.

As to nasal mucosa atrophy, it was found in 23 patients, nine LL, four BL, four BT, three BB and three NP, which may be present from the onset of the disease or after the remission of the infiltration process, in which the atrophy is justified by dryness of mucosa glands and marked vasculitis (Photo 4) found in 67 patients (38.7%).

We could observe that vasculitis leads to reduction of blood circulation, a fact that made us raise the hypothesis that its intervention on the action of polychemotherapic treatment in the nasal mucosa could enable bacillus viability with progression of nasal lesions; in addition, there is persistence in the mucosa even after polychemotherapy, which is observed in patients with reactions types I, II or Neural years after treatment and comprising, with it, a new theory for the conduction of future studies in the area.

## CONCLUSION

Because the nose is considered the entry and exit point of *Mycobacterium lepra*e, we would like to attract the attention of healthcare specialists, specially Otorhinolaryngologists, to suspect of Leprosy in patients who have nasal lesions with infiltration, crusts and vasculitis, because we found mucosa affections in all patients seen in this study. The identification of mucosa lesions using rigid or flexible endoscopy provides further accuracy and early diagnosis, and the Otorhinolaryngologist is indispensable to work with the multidisciplinary team for follow-up and treatment of patients, preventing the sequelae that contribute to disease stigma and social exclusion.
